# Rib Fixation for Multiple Rib Fractures: Healthcare Professionals Perceived Barriers and Facilitators to Clinical Implementation

**DOI:** 10.1007/s00268-023-06973-y

**Published:** 2023-04-04

**Authors:** Inge Spronk, Suzanne F. M. Van Wijck, Esther M. M. Van Lieshout, Michael H. J. Verhofstad, Jonne T. H. Prins, Mathieu M. E. Wijffels, Suzanne Polinder, the FixCon Study Group, Taco J. Blokhuis, Erik R. De Loos, Elvira R. Flikweert, Albert F. Pull ter Gunne, Akkie N. Ringburg, W. Richard Spanjersberg, Gerben Van der Bij, Floortje C. Van Eijck, Pieter J. Van Huijstee, Gust Van Montfort, Jefrey Vermeulen, Dagmar I. Vos

**Affiliations:** 1 Department of Public Health, Erasmus MC, University Medical Center Rotterdam, Doctor Molewaterplein 40, P.O. Box 2040, 3015 GD Rotterdam, The Netherlands; 2 Trauma Research Unit, Department of Surgery, Erasmus MC, University Medical Center Rotterdam, Doctor Molewaterplein 40, 3015 GD Rotterdam, The Netherlands

## Abstract

**Background:**

Surgical stabilization of rib fractures (SSRF) is associated with improved respiratory symptoms and shorter intensive care admission in patients with flail chest. For multiple rib fractures, the benefit of SSRF remains a topic of debate. This study investigated barriers and facilitators of healthcare professionals to SSRF as treatment for multiple traumatic rib fractures.

**Methods:**

Dutch healthcare professionals were asked to complete an adapted version of the Measurement Instrument for Determinants of Innovations questionnaire to identify barriers and facilitators of SSRF. If ≥ 20% of participants responded negatively, the item was considered a barrier, and if ≥ 80% responded positively, the item was considered a facilitator.

**Results:**

Sixty-one healthcare professionals participated; 32 surgeons, 19 non-surgical physicians, and 10 residents. The median experience was 10 years (P_25_–P_75_ 4–12). Sixteen barriers and two facilitators for SSRF in multiple rib fractures were identified. Barriers included lack of knowledge, experience, evidence on (cost-)effectiveness, and the implication of more operations and higher medical costs. Facilitators were the assumption that SSRF alleviates respiratory problems and the feeling that surgeons are supported by colleagues for SSRF. Non-surgeons and residents reported more and several different barriers than surgeons (surgeons: 14; non-surgical physicians: 20; residents: 21; *p* < 0.001).

**Conclusion:**

For adequate implementation of SSRF in patients with multiple rib fractures, implementation strategies should address the identified barriers. Especially, improved clinical experience and scientific knowledge of healthcare professionals, and high-level evidence on the (cost-) effectiveness of SSRF potentially increase its use and acceptance.

**Supplementary Information:**

The online version contains supplementary material available at 10.1007/s00268-023-06973-y.

## Introduction

Rib fractures occur in 10–39% of patients with blunt chest trauma, accounting for 10% of all trauma admissions [1–3]. Rib fractures are associated with morbidity and long-term impaired quality of life [1, 4–6]. A typical pattern of multiple fractures is a flail chest, which is defined as three or more consecutive ribs fractured in at least two places [7, 8]. Other fracture patterns with three or more fractured ribs are often called multiple rib fractures.

Increasingly, surgical stabilization of rib fractures (SSRF) of a flail chest shows superior results over nonoperative treatment [9–12]. For patients with multiple rib fractures without a flail chest, particularly low-level evidence suggests that SSRF results in shorter Intensive Care Unit (ICU) and hospital stays, lower rates of pneumonia and empyema, reduced need for opioid analgesics and mechanical ventilation, and earlier return to work and social activities [13–16]. Currently, a multicenter randomized controlled trial investigates the effects of SSRF versus nonoperative treatment of multiple rib fractures in patients without a flail chest (FixCon trial) [17].

Concomitantly to growing evidence, an implementation strategy should be developed to successfully implement SSRF as a treatment for multiple rib fractures [18]. Barriers and facilitators possibly influencing the implementation of SSRF should be identified, such as experience, knowledge, and available resources, to ensure that the implementation strategy includes relevant determinants, is feasible, and is tailored to the context [19]. Therefore, this study identified barriers and facilitators of healthcare professionals (HCPs) to SSRF for treatment of trauma patients with multiple rib fractures. The secondary aim was to compare barriers and facilitators between groups of HCPs.

## Material and methods

### Study design, setting, and participants

This cross-sectional questionnaire study was reported following the ‘Checklist for Reporting of Survey Studies’ (CROSS) (Online Resource 1) [20]. This study was conducted alongside the earlier described FixCon trial [17]. The online questionnaire was programmed in LimeSurvey (Version 2.06lts) [21] and disseminated via the project team and FixCon [17] study group to surgeons (trauma/thoracic/general surgeons), non-surgical physicians (intensivists, pulmonologists, anesthetists, rehabilitation specialists), and residents involved in treatment of adults with rib fractures after blunt trauma from Dutch hospitals that do and do not participate in the FixCon trial. Recipients were encouraged to forward it to colleagues. The survey platform registered IP addresses to prevent submitting more than one response. Data were collected between April 8 and August 31, 2021.

### Questionnaire

The Measurement Instrument for Determinants of Innovations (MIDI) was used [19, 22]. MIDI identifies barriers and facilitators of implementation and guides the development of implementation strategies in healthcare settings. MIDI contains 29 items that should be adapted to the specific setting [22]. For this study, items were adapted to identify barriers and facilitators to the extended indication for SSRF. Twenty MIDI items (transposed in 44 questions) were included, two items of the Barriers and Facilitators Assessment Instrument (BFAI) [23], and 13 items that were developed after consultation with nine HCPs involved in SSRF (Online Resource 2). Questions covered determinants on the indication for SSRF for multiple rib fractures; concept and experience; perceived (dis)advantages; organizational aspects; other potential barriers. Each question had five response options, ranging from ‘totally disagree’ (1) to ‘totally agree’ (5). One additional open-ended question asked about any other perceived barriers. These responses were analyzed qualitatively. Furthermore, HCP characteristics were requested, including sex, age, years of clinical experience, and specialty. It was also asked whether their hospital participated in the FixCon trial and if SSRF was routinely performed in their hospital.

### Statistical analyses

Questionnaire responses were anonymously analyzed in SPSS version 25.0 (SPSS, Chicago, Ill., USA). Continuous variables were reported as median and quartiles, and categorical variables as numbers with percentages. Conform previous studies [24–26], positively worded statements to which ≥ 20% of the HCPs responded ‘(totally) disagree’ were considered barriers. Positively worded statements to which ≥ 80% of the HCPs responded ‘(totally) agree’ were considered facilitators. For negatively worded statements the opposite was applied.

Fisher exact tests were used to compare barriers and facilitators between subgroups. Subgroup comparisons included: (1) Surgeons versus non-surgical physicians versus residents; and (2) Surgeons from a FixCon trial center versus surgeons from a non-FixCon trial center.

## Results

Sixty-one HCPs participated: 32 surgeons, 19 non-surgical physicians, and 10 residents with various backgrounds (Table 1). Most HCPs were aged 36–45 years (n = 28; 46%). The majority were male (n = 51; 84%), and most were (trauma) surgeons (n = 28; 46%). The median clinical experience was 10 years (P_25_–P_75_ 6–14) for (trauma) surgeons, 10 years (P_25_–P_75_ 5–15) for non-surgical physicians, and 0 years (P_25_–P_75_ 0–4) for residents About half of the HCPs worked in a hospital participating in the FixCon trial (n = 33; 54%). Twenty-four (39%) respondents performed SSRF themselves, whereas for 29 (48%) respondents, a colleague performed SSRF.

**Table 1:** Participant characteristics

	Total (n = 61)
Sex (male)	51 (84%)
*Age (years)*	
18–35	11 (18%)
36–45	28 (46%)
46–55	19 (31%)
> 55	3 (5%)
*Specialty*	
(Trauma) surgeon	29 (49%)
Thoracic surgeon^a^	3 (5%)
Intensivist	4 (7%)
Pulmonologist	4 (7%)
Anesthetist	10 (16%)
Rehabilitation specialist	1 (2%)
Resident	10 (16%)
Experience (years)	10.0 (3.5–12.0)
FixCon trial center^b^	33 (54%)
(Trauma) surgeon	14 (44%)
*Hospital performing SSRF*	
I perform SSRF	24 (39%)
My colleagues perform SSRF	29 (48%)
No	5 (8%)
Not sure	3 (5%)

Data are shown as n (%) or as median (P_25_-P_75_)

SSRF, surgical stabilization of rib fractures

^a^Please note that in the Netherlands, the specialty ‘thoracic surgery’ can include both cardiac and lung surgery

^**b**^Hospitals that participate in the multicenter FixCon trial

### Barriers and facilitators to SSRF

Overall, 16 barriers and two facilitators were identified (Table 2 and Online Resource 2) indicating a negative attitude towards implementing SSRF for multiple rib fractures without a flail chest. Major differences in barriers and facilitators between surgeons, non-surgical physicians, and residents were revealed (Table 3 and Online Resource 3). Twenty determinants were statistically significantly different among the three subgroups. Non-surgical physicians and residents reported more barriers than surgeons (surgeons: 14; non-surgical physicians: 20; residents: 21, *p* < 0.001). Furthermore, surgeons reported seven and residents six facilitators, while non-surgical physicians reported none. Surgeons working in a hospital participating in the FixCon trial reported fewer barriers (n = 15 vs. n = 19, *p* < 0.001), but also fewer facilitators (n = 7 vs. n = 10, *p* < 0.001) than surgeons working in a non-FixCon trial center. Only eight barriers and six facilitators overlapped, and eight determinants differed statistically significantly between the FixCon and non-FixCon trial centers (Table 4 and Online Resource 3).

**Table 2:**
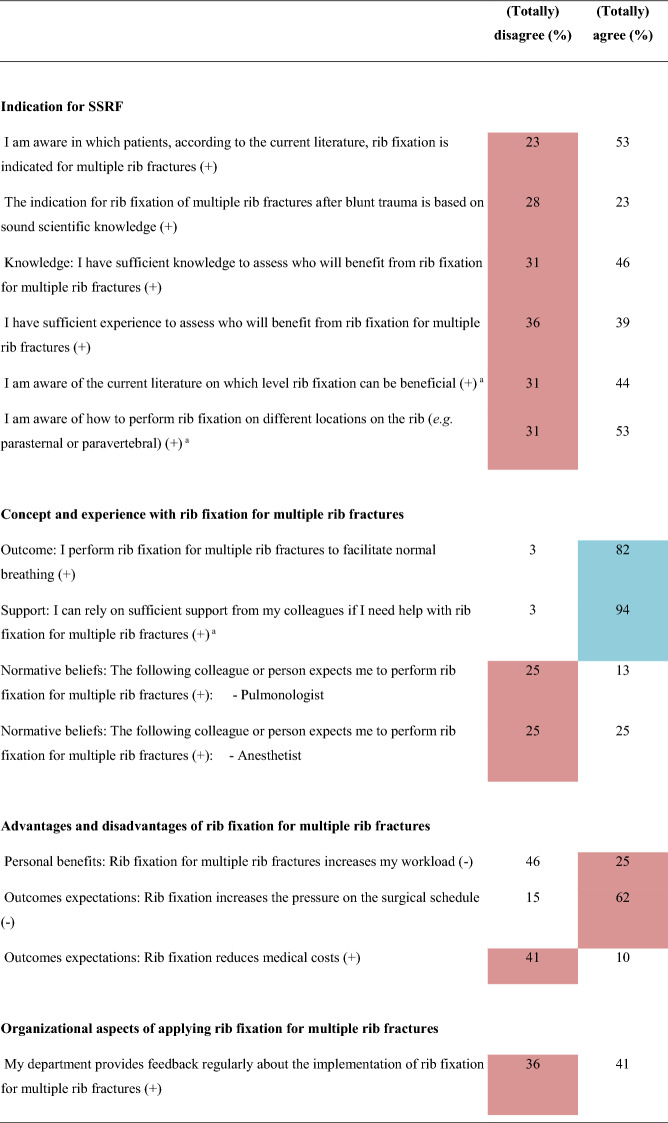

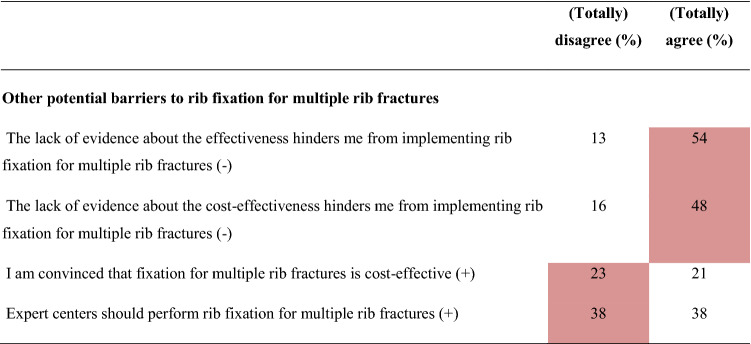
Summary of barriers and facilitators influencing the implementation of surgical stabilization of rib fractures for multiple rib fractures after blunt trauma in adults (n = 61)

( +) indicates positive statement; (-) indicates negative statement. Data are shown as percentages. Barriers are highlighted in red; facilitators are highlighted in blue

^*^Indicates that the question applies exclusively to surgeons, therefore only the surgeon’s responses are displayed

**Table 3:**
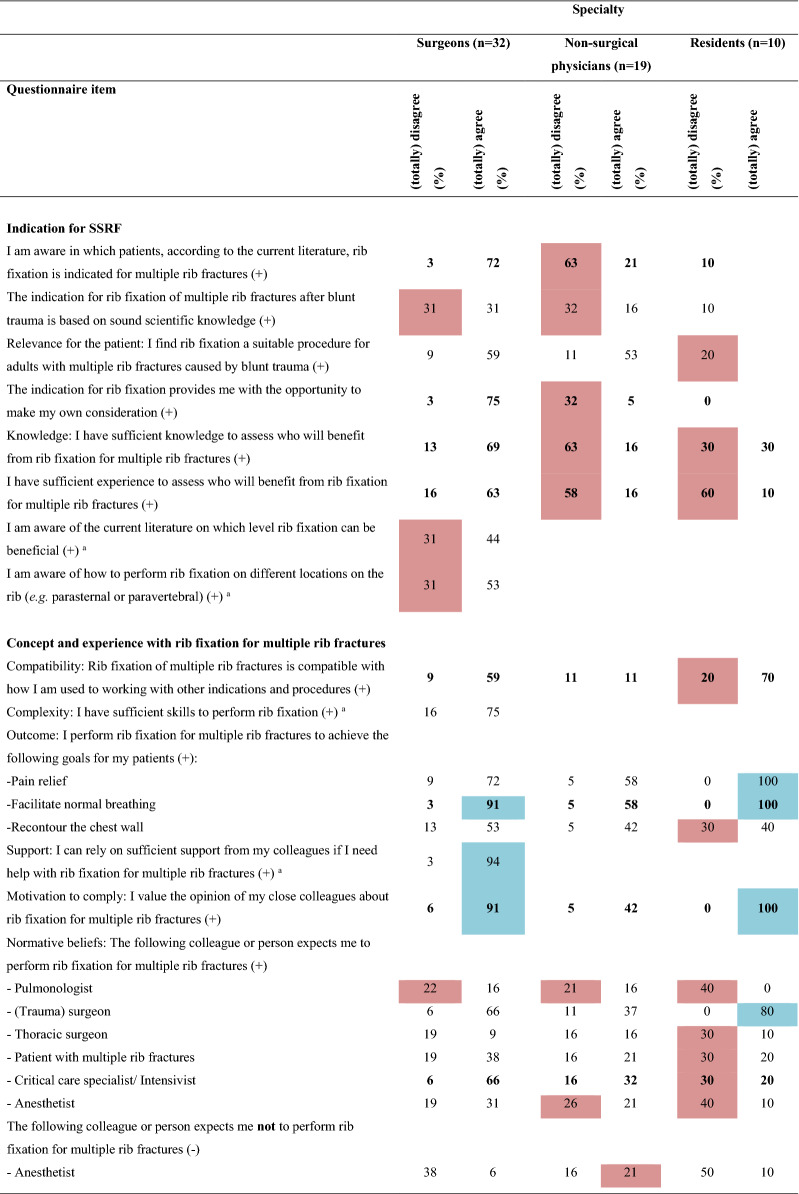

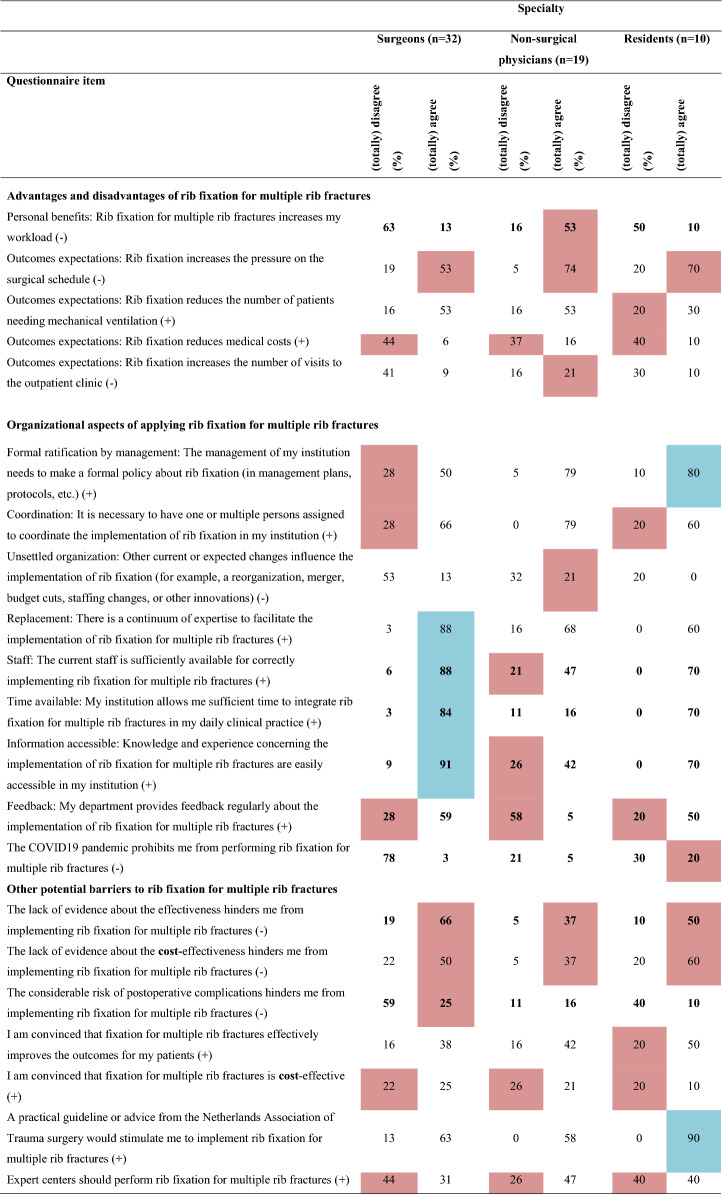
Comparison of barriers and facilitators influencing the implementation of surgical stabilization of rib fractures for multiple rib fractures after blunt trauma in adults between healthcare providers from different specialties

( +) indicates positive statement; (-) indicates negative statement. Data are shown as percentages. Barriers are highlighted in red; facilitators are highlighted in blue. Bold numbers indicate statistically significant differences between subgroups

^a^Indicates that the question applies exclusively to surgeons

**Table 4:**
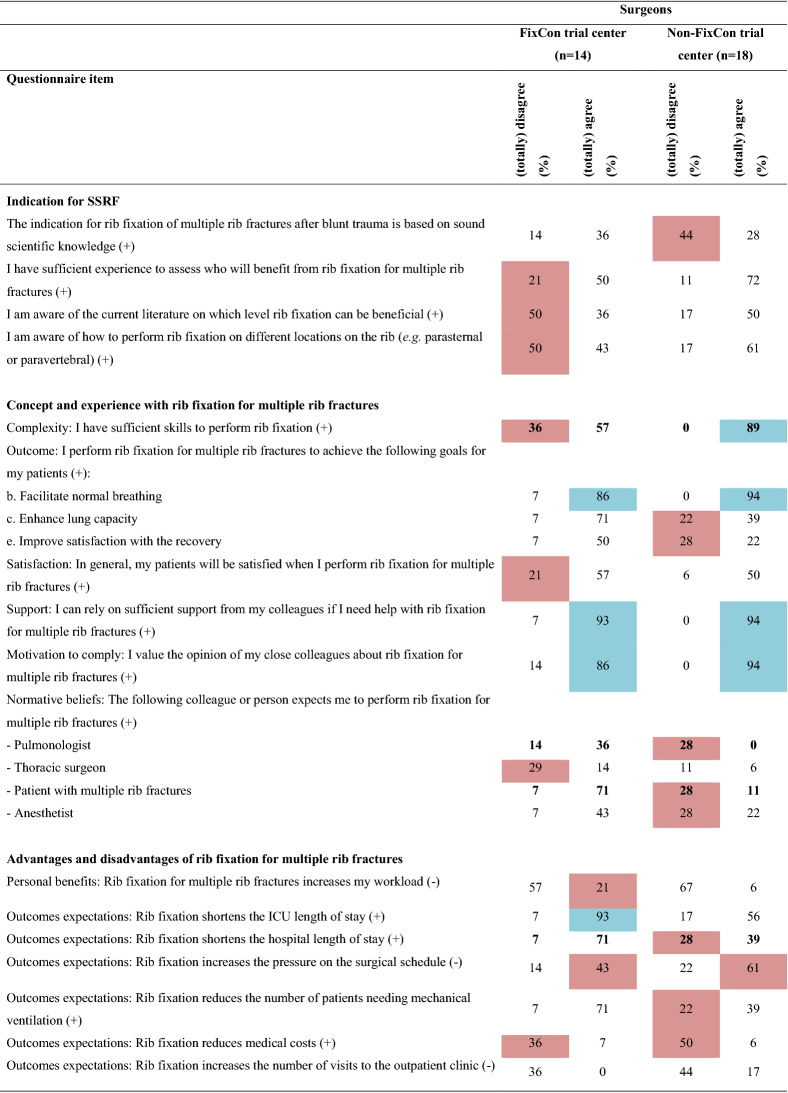

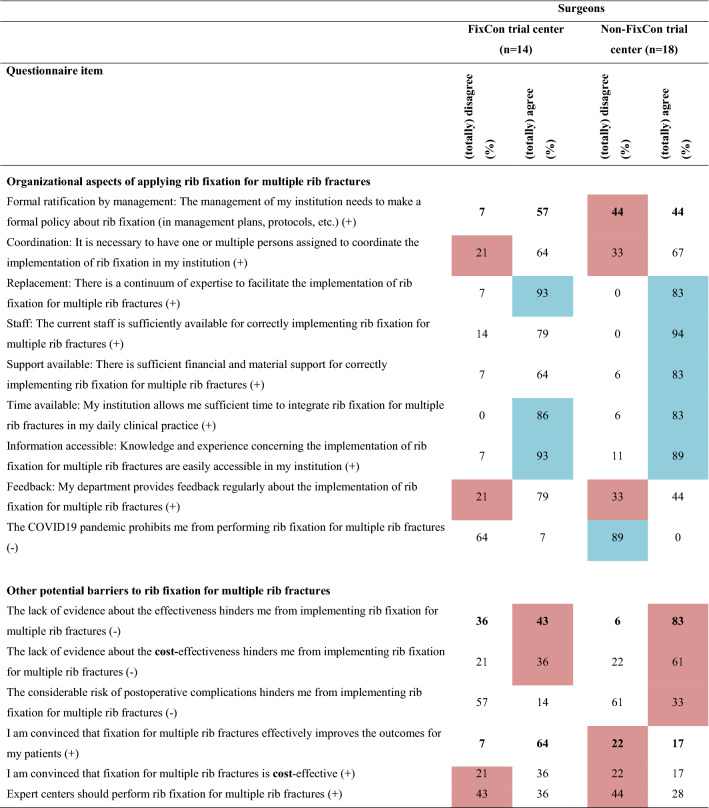
Comparison of barriers and facilitators influencing the implementation of surgical stabilization of rib fractures for multiple rib fractures after blunt trauma in adults between surgeons from centers participating and centers not participating in the FixCon trial

( +) indicates positive statement; (-) indicates negative statement. Data are shown as percentages. Barriers are highlighted in red; facilitators are highlighted in blue. Bold numbers indicate statistically significant differences between subgroups

#### Indication for SSRF

For setting the indication for SSRF, six barriers and no facilitators were identified (Table 2 and Online Resource 2). Most frequently, HCPs indicated that they are too inexperienced (n = 22; 36%) and have insufficient knowledge (n = 19; 31%) about which patient will benefit from SSRF, and the rib level (n = 10; 31%) and location on the rib (n = 10; 31%) where SSRF could be beneficial. They also lacked belief that SSRF is based on sound scientific knowledge (n = 17; 28%) and lacked awareness of for whom rib fixation is indicated (n = 14; 23%). Subgroup analysis demonstrated that surgeons did not indicate inexperience, insufficient knowledge, or unawareness as a barrier to indication setting for SSRF (Table 3 and Online Resource 3).

#### Concept and experience

Two barriers and two facilitators were identified for concept and experience with SSRF. The goal to promote normal breathing with SSRF was a facilitator (n = 50; 82%). Solely for surgeons, a facilitator was that they can rely on sufficient support from colleagues (n = 30; 94%). Respondents assumed that pulmonologists (n = 15; 25%) and anesthetists (n = 15; 25%) do not consider SSRF as a treatment option for patients with multiple rib fractures.

#### Perceived (dis)advantages

Three barriers were perceived as disadvantages: the assumed increases of pressure on the surgical schedule (n = 38; 62%), medical costs (n = 25; 41%), and workload (n = 15; 25%). Interestingly, subgroup analysis revealed that only the non-surgical physicians perceived the increased workload as a barrier (surgeons:13%; non-surgical physicians: 53%; residents: 10%; *p* = 0.001).

#### Organizational aspects

One organizational aspect was a barrier: limited regular feedback from their department on the application of SSRF (n = 22; 36%). Remarkably, availability of staff (surgeons: 6%; non-surgical physicians: 21%; residents: 0%; *p* = 0.012) and easy access in the organization to knowledge and experience concerning the implementation of SSRF (surgeons: 9%; non-surgical physicians: 26%; residents: 0%; *p* < 0.001) were facilitators for surgeons, but barriers for non-surgical physicians. Surgeons also indicated time available to integrate SSRF in their organization was a facilitator, in contrast to the non-surgical physicians and residents (surgeons: 84%; non-surgical physicians: 16%; residents: 70%, *p* < 0.001).

#### Other barriers

Four other barriers were reported: lack of evidence about effectiveness (n = 33; 54%) and cost-effectiveness (n = 29; 48%); the belief that SSRF is not cost-effective (n = 14; 23%); and having expert centers for SSRF would be a barrier (n = 23; 38%). Surgeons, in contrast to other HCPs, implied that a considerable risk of postoperative complications is a barrier (surgeons: 25%; non-surgical physicians: 16%; residents: 10%; *p* < 0.001). Residents indicated that a practical guideline or advice from the national trauma surgery association would be a facilitator (90%).

The open-ended question on other potential barriers was answered by 31 (51%) respondents. Most responses (n = 14; 45%) were about the lack of evidence about the (cost-)effectiveness and the indication of SSRF for multiple rib fractures (n = 4; 13%). Other concerns were about logistical factors including the operating room schedule (n = 4; 13%) and the availability of local expertise (n = 3; 10%).

#### FixCon versus non-fixCon trial centers

Responses differed between surgeons working in FixCon and non-FixCon trial centers (Table 4). Having sufficient skills to perform SSRF was a barrier for FixCon trial surgeons, but a facilitator for those not working in a FixCon trial center (barrier: 36 vs. 0%; *p* = 0.024). SSRF was expected to increase the hospital length of stay by non-FixCon surgeons, but not by FixCon surgeons (28% vs. 7%; *p* = 0.038). Barriers to non-FixCon surgeons were that pulmonologists (28 vs. 14%; *p* = 0.023) and patients (28 vs. 14%; *p* = 0.003) do not expect them to perform SSRF; absence of a formal policy about SSRF (*e.g.* in protocols) (44 vs. 7%; *p* = 0.042); and they were not convinced that SSRF for multiple rib fractures effectively improves outcomes (22 vs. 7%; *p* = 0.027). The lack of evidence about the effectiveness of SSRF was a stronger barrier for non-FixCon surgeons than for surgeons from a FixCon trial center (83 vs. 43%; *p* = 0.041).

## Discussion

Sixteen barriers and two facilitators for implementing SSRF were identified. Most barriers concerned lacking scientific evidence for the indication and (cost-)effectiveness of SSRF. Surgeons perceived fewer barriers and more facilitators than non-surgical physicians and residents; especially in the workload and organizational aspects. Facilitators were the assumption that SSRF alleviates respiratory problems, and for surgeons, the feeling that they are supported by colleagues for SSRF. Barriers and facilitators differed between surgeons from centers that do and do not participate in the FixCon trial. Differences consisted mostly of the extent to which they were convinced that patients with multiple rib fractures benefit from SSRF; *e.g.*, surgeons from non-FixCon trial centers expected SSRF to increase the length of hospital stay, contrasting evidence showing that SSRF is associated with equal or shorter hospital length of stay [12, 27].

To the best of our knowledge, this is the first study investigating barriers and facilitators to implementing SSRF for trauma patients with multiple rib fractures including surgeons and non-surgeons. A previous study surveyed 450 surgeons in 2007 for their opinion about SSRF and sternal fracture repair [28]. Many respondents reported that the scientific literature was insufficient for the indication for SSRF, which is similar to our findings. Interestingly, a large majority of respondents in that survey stated that they did not know any published randomized trials about SSRF. Although many studies about SSRF have been published since then, this unawareness of the scientific literature for the indication of SSRF could also have played a role in our study. Several studies have described barriers and facilitators for implementing rib fracture management protocols [29–31]. For example, a dedicated rib fracture consultation service was started when it was realized that many trauma patients who could have benefitted from SSRF were missed [29]. Consequently, embedding consultation with a team of dedicated surgeons who regularly perform SSRF was included in the care of patients with rib fractures. Insufficient knowledge, experience, and availability of staff were considered barriers to SSRF, which is similar to the barriers found in the current study. Another study showed that an important facilitator for an analgesic protocol for rib fractures patients was the practical fact that starting the new protocol became the least labor-intensive method for initiating patient care upon admission [31].

Although not for SSRF specifically, several barriers and facilitators were described for the implementation of a chest injury care bundle, including a standard set of interventions for patients with chest injuries [32]. HCPs identified that new interventions must be evidence-based, easy to follow, and easily accessible [32]. Also, the belief that the intervention improves patient care and the support from colleagues was considered very important. Our findings share these themes.

A strategy for implementing SSRF for patients with multiple rib fractures is most likely to succeed when it enhances the identified facilitators and focuses on diminishing the barriers [18, 33, 34]. Not surprisingly, the most important barrier to implementation was the lack of evidence. At the time of the survey, there was a paucity of data from clinical trials about SSRF in patients with multiple rib fractures without a flail chest. However, several trials were recently published [12, 35] and more are expected soon [17, 36]. With increasing scientific evidence for the (cost-)effectiveness of SSRF becoming available, the barrier related to lack of evidence will likely diminish. Nevertheless, as the field of implementation science has shown multiple times, sound scientific evidence alone does not guarantee uptake [18, 33, 34]. Likely, disseminating SSRF also relies heavily on the opinion and practices of colleagues and mentors. Our results demonstrate that surgeons perceived the fewest barriers in knowledge and experience, and generally felt most facilitated by their close colleagues and their organization. An implementation strategy is likely to be most successful when it allows surgeons to disseminate their knowledge and experience to their colleagues, residents, and non-surgical colleagues in the anesthesia and pulmonology departments. As for organizational aspects, the strategy should focus on optimizing the planning of SSRF in a busy operation room schedule and providing regular feedback about the implementation of SSRF to all HCPs involved.

This study has several strengths and limitations. A strength includes the use of the widely-used MIDI to identify barriers and facilitators. Another strength is the diversity of HCPs that completed the questionnaire which allowed subgroup analyses. However, non-surgical physicians could potentially have confounded the overall results concerning aspects more specific to surgery. Another limitation includes that some respondents were invited via the FixCon trial network, who might be more positive about SSRF because of their connection with a surgeon who is involved in a clinical trial specifically studying SSRF for multiple rib fractures. Because our questionnaire study was performed anonymously and with an open link, we have no insight into the response rate and we were unable to determine whether non-responders differed from responders. Also, although IP addresses were registered, double submissions could theoretically not be completely prevented. The study is limited by the relatively small number (n = 61) of respondents, which raises the suspicion that the non-responders were less interested and therefore less positive about SSRF in general than the respondents. In addition, most respondents (87%) worked in a center that already performs SSRF. Potentially, the results of this study are not directly generalizable to HCPs working in a center that does not have an established SSRF program. As a final limitation, 16% of the respondents were residents with relatively limited experience, which might have affected their insights into barriers and facilitators. Despite these limitations, the questionnaire results provide important insights to develop implementation strategies. However, potentially other not surveyed factors could become apparent later in the implementation process. In the end, changing behavior is a complex process, for which an implementation strategy provides a starting point. Naturally, when progressing in the implementation process, continuing awareness for identifying new barriers and facilitators is warranted.

In conclusion, successfully implementing SSRF for trauma patients with multiple rib fractures requires growing scientific and clinical knowledge. Besides this, developing implementation strategies for SSRF for multiple rib fractures should aim at overcoming the other barriers and enhancing the facilitators identified in this study.

## Supplementary Information

Below is the link to the electronic supplementary material.

Supplementary file1 (DOCX 16 kb)

Supplementary file2 (DOCX 27 kb)

Supplementary file3 (DOCX 43 kb)
