# Supplementary material for: Rib Fixation for Multiple Rib Fractures: Healthcare Professionals Perceived Barriers and Facilitators to Clinical Implementation
Source: World J Surg. 2023 Apr 4;47(7):1692–703. doi: 10.1007/s00268-023-06973-y (PMC10229739; doi:10.1007/s00268-023-06973-y)
Supplement: Supplementary file 1 — Supplementary file1 (DOCX 16 kb) [file 268_2023_6973_MOESM1_ESM.docx]

**Online Resource 1. Completed ‘Checklist for Reporting of Survey Studies (CROSS)’**

| **Section/topic** | **Page number in manuscript** |
| --- | --- |
| Title and abstract | Title page; abstract document |
| Background | 1 |
| Purpose/aim | 1 |
| Study design | 2 |
| Data collection method | 2-3 |
| Sample characteristics | 2-3 |
| Survey administration | 3 |
| Study preparation | 2-3 |
| Ethical considerations | 2 |
| Statistical analysis | 3 |
| Respondent characteristics | 4 |
| Descriptive results | 4-7 |
| Main findings | 4-8 |
| Limitations | 9-10 |
| Interpretations | 9-10 |
| Generalizability | 9-10 |
| Role of the funding source | Title page |
| Conflict of interest | Title page |
| Acknowledgements | Not applicable |

Manuscript title: Rib fixation for multiple rib fractures: healthcare professionals perceived barriers and facilitators to clinical implementation

Journal: World Journal of Surgery

Authors: Inge Spronk PhD, Suzanne F.M. Van Wijck MD, Esther M.M. Van Lieshout PhD MSc, Michael H.J. Verhofstad MD PhD, Jonne T.H. Prins MD PhD, Mathieu M.E. Wijffels MD PhD, Suzanne Polinder PhD (on behalf of the FixCon study group)

Correspondence: Inge Spronk, Erasmus MC, Department of Public Health, i.spronk@erasmusmc.nl
