# Supplementary material for: Rib Fixation for Multiple Rib Fractures: Healthcare Professionals Perceived Barriers and Facilitators to Clinical Implementation
Source: World J Surg. 2023 Apr 4;47(7):1692–703. doi: 10.1007/s00268-023-06973-y (PMC10229739; doi:10.1007/s00268-023-06973-y)
Supplement: Supplementary file 2 — Supplementary file2 (DOCX 27 kb) [file 268_2023_6973_MOESM2_ESM.docx]

# **Online Resource 2. Survey responses for the implementation of surgical stabilization of rib fractures for multiple rib fractures after blunt trauma in adults (n=61)**

| **No.** | **Origin item** | **Questionnaire item** | **Disagree/ totally disagree (%)** | **Neutral (%)** | **Agree/ totally agree (%)** |
| --- | --- | --- | --- | --- | --- |
|  | **Indication for SSRF** | | | | |
| 1 |  | I am aware in which patients, according to the current literature, rib fixation is indicated for multiple rib fractures (+) | 23.0 | 24.6 | 52.5 |
| 2 | MIDI 2 | The indication for rib fixation of multiple rib fractures after blunt trauma is based on sound scientific knowledge (+) | 27.9 | 49.2 | 23.0 |
| 3 | MIDI 7 | Relevance for the patient: I find rib fixation a suitable procedure for adults with multiple rib fractures caused by blunt trauma (+) | 11.5 | 36.1 | 52.5 |
| 4 | BFAI 1 | The indication for rib fixation provides me with the opportunity to make my own consideration (+) | 11.5 | 36.1 | 52.5 |
| 5 | BFAI 2 | The indication for rib fixation provides me with the opportunity to include the values of the patient (+) | 13.1 | 44.3 | 42.6 |
| 6 | MIDI 17 | Knowledge: I have sufficient knowledge to assess who will benefit from rib fixation for multiple rib fractures (+) | 31.1 | 23.0 | 45.9 |
| 7 |  | I have sufficient experience to assess who will benefit from rib fixation for multiple rib fractures (+) | 36.1 | 24.6 | 39.3 |
| 8 |  | I am aware of the current literature on which level rib fixation can be beneficial (+) ^a^ | 31.3 | 24.9 | 43.8 |
| 9 |  | I am aware of how to perform rib fixation on different locations on the rib (e.g. parasternal or paravertebral) (+) ^a^ | 31.3 | 15.6 | 53.1 |
|  | **Concept and experience with rib fixation** **for multiple rib fractures** | |  |  |  |
| 10 | MIDI 3 | Completeness: The manufacturer’s rib fixation hardware provides the necessary operative instructions and materials (+) ^a^ | 3.1 | 34.4 | 62.5 |
| 11 | MIDI 5 | Compatibility: Rib fixation of multiple rib fractures is compatible with how I am used to working with other indications and procedures (+) | 11.5 | 42.6 | 45.9 |
| 12 | MIDI 4 | Complexity: I have sufficient skills to perform rib fixation (+) ^a^ | 15.6 | 9.4 | 75.0 |
|  | MIDI 9A | Outcome: I perform rib fixation for multiple rib fractures to achieve the following goals for my patients (+): |  |  |  |
| 13 |  | Pain relief | 6.6 | 21.3 | 72.1 |
| 14 |  | Facilitate normal breathing | 3.3 | 14.8 | 82.0 |
| 15 |  | Enhance lung capacity | 13.1 | 42.6 | 44.3 |
| 16 |  | Recontour the chest wall | 13.1 | 39.3 | 47.5 |
| 17 |  | Improve satisfaction with the recovery | 11.5 | 45.9 | 42.6 |
| 18 | MIDI 11 | Satisfaction: In general, my patients will be satisfied when I perform rib fixation for multiple rib fractures (+) | 9.8 | 57.4 | 32.8 |
| 19 | MIDI 13 | Support: I can rely on sufficient support from my colleagues if I need help with rib fixation for multiple rib fractures (+) ^a^ | 3.1 | 3.1 | 93.8 |
| 20 | MIDI 15b | Motivation to comply: I value the opinion of my close colleagues about rib fixation for multiple rib fractures (+) | 4.9 | 18.0 | 77.0 |
|  | MIDI 15a | Normative beliefs: The following colleague or person expects me to perform rib fixation for multiple rib fractures (+) |  |  |  |
| 21 |  | - Pulmonologist | 24.6 | 62.3 | 13.1 |
| 22 |  | - (Trauma) surgeon | 6.6 | 34.3 | 59.0 |
| 23 |  | - Thoracic surgeon | 19.7 | 68.9 | 11.5 |
| 24 |  | - Patient with multiple rib fractures | 19.7 | 50.8 | 29.5 |
| 25 |  | - Critical care specialist/ Intensivist | 13.1 | 39.3 | 47.5 |
| 26 |  | - Anesthetist | 24.6 | 50.8 | 24.6 |
|  |  | The following colleague or person expects me **not** to perform rib fixation for multiple rib fractures (-) |  |  |  |
| 27 |  | - Pulmonologist | 27.9 | 62.3 | 9.8 |
| 28 |  | - (Trauma) surgeon | 39.3 | 49.2 | 11.5 |
| 29 |  | - Thoracic surgeon | 34.4 | 55.7 | 9.8 |
| 30 |  | - Patient with multiple rib fractures | 36.1 | 57.4 | 6.6 |
| 31 |  | - Critical care specialist/ Intensivist | 36.1 | 54.1 | 9.8 |
| 32 |  | - Anesthetist | 32.8 | 55.7 | 11.5 |
|  | Advantages and disadvantages of **rib fixation** **for multiple rib fractures** | |  |  |  |
| 33 | MIDI 8a | Personal benefits: Rib fixation for multiple rib fractures supports me to provide better care for my patients (+) | 6.6 | 39.3 | 54.1 |
| 34 | MIDI 8b | Personal benefits: Rib fixation for multiple rib fractures increases my workload (-) | 45.9 | 29.5 | 24.6 |
| 35 | MIDI 9b | Outcomes expectations: Rib fixation shortens the ICU length of stay (+) | 13.1 | 21.3 | 65.6 |
| 36 | MIDI 9c | Outcomes expectations: Rib fixation shortens the hospital length of stay (+) | 14.8 | 27.9 | 57.4 |
| 37 | MIDI 9d | Outcomes expectations: Rib fixation increases the pressure on the surgical schedule (-) | 14.8 | 23.0 | 62.3 |
| 38 | MIDI 9e | Outcomes expectations: Rib fixation reduces the number of patients needing mechanical ventilation (+) | 16.4 | 34.3 | 49.2 |
| 39 | MIDI 9f | Outcomes expectations: Rib fixation reduces pulmonary complications (+) | 13.1 | 27.9 | 59.0 |
| 40 | MIDI 9g | Outcomes expectations: Rib fixation improves the quality of life of the patient (+) | 9.8 | 44.3 | 45.9 |
| 41 | MIDI 9h | Outcomes expectations: Rib fixation reduces medical costs (+) | 41.0 | 49.2 | 9.8 |
| 42 | MIDI 9i | Outcomes expectations: Rib fixation increases the number of visits to the outpatient clinic (-) | 31.1 | 55.7 | 13.1 |
|  | **Organizational aspects of applying rib fixation** **for multiple rib fractures** | |  |  |  |
| 43 | MIDI 19 | Formal ratification by management: The management of my institution needs to make a formal policy about rib fixation (in management plans, protocols, etc.) (+) | 18.0 | 18.0 | 63.9 |
| 44 | MIDI 25 | Coordination: It is necessary to have one or multiple persons assigned to coordinate the implementation of rib fixation in my institution (+) | 18.0 | 13.1 | 68.9 |
| 45 | MIDI 26 | Unsettled organization: Other current or expected changes influence the implementation of rib fixation (for example, a reorganization, merger, budget cuts, staffing changes, or other innovations) (-) | 41.0 | 45.9 | 13.1 |
| 46 | MIDI 20 | Replacement: There is a continuum of expertise to facilitate the implementation of rib fixation for multiple rib fractures (+) | 6.6 | 16.4 | 77.0 |
| 47 | MIDI 21 | Staff: The current staff is sufficiently available for correctly implementing rib fixation for multiple rib fractures (+) | 9.8 | 18.0 | 72.1 |
| 48 | MIDI 22 | Support available: There is sufficient financial and material support for correctly implementing rib fixation for multiple rib fractures (+) | 4.9 | 39.3 | 55.7 |
| 49 | MIDI 23 | Time available: My institution allows me sufficient time to integrate rib fixation for multiple rib fractures in my daily clinical practice (+) | 4.9 | 34.4 | 60.7 |
| 50 | MIDI 27 | Information accessible: Knowledge and experience concerning the implementation of rib fixation for multiple rib fractures are easily accessible in my institution (+) | 13.1 | 14.8 | 72.1 |
| 51 | MIDI 28 | Feedback: My department provides feedback regularly about the implementation of rib fixation for multiple rib fractures (+) | 36.1 | 23.0 | 41.0 |
| 52 |  | The COVID19 pandemic prohibits me from performing rib fixation for multiple rib fractures (-) | 52.5 | 41.0 | 6.6 |
|  | **Other potential barriers to rib fixation** **for multiple rib fractures** | |  |  |  |
| 53 |  | The lack of evidence about the effectiveness hinders me from implementing rib fixation for multiple rib fractures (-) | 13.1 | 32.8 | 54.1 |
| 54 |  | The lack of evidence about the **cost-**effectiveness hinders me from implementing rib fixation for multiple rib fractures (-) | 16.4 | 36.1 | 47.5 |
| 55 |  | The considerable risk of postoperative complications hinders me from implementing rib fixation for multiple rib fractures (-) | 41.0 | 39.3 | 19.7 |
| 56 |  | I am convinced that fixation for multiple rib fractures effectively improves the outcomes for my patients (+) | 16.4 | 42.6 | 41.0 |
| 57 |  | I am convinced that fixation for multiple rib fractures is **cost**-effective (+) | 23.0 | 55.7 | 21.3 |
| 58 |  | A practical guideline or advice from the Netherlands Association of Trauma surgery would stimulate me to implement rib fixation for multiple rib fractures (+) | 6.6 | 27.9 | 65.6 |
| 59 |  | Expert centers should perform rib fixation for multiple rib fractures (+) | 37.7 | 24.6 | 37.7 |

Note: (+) indicates positive statement; (-) indicates negative statement. Data are shown as percentages. Barriers are highlighted in red; facilitators are highlighted in blue.
^a^ Indicates that the question applies exclusively to surgeons, therefore only the surgeon’s responses are displayed.

Manuscript title: Rib fixation for multiple rib fractures: healthcare professionals perceived barriers and facilitators to clinical implementation

Journal: World Journal of Surgery

Authors: Inge Spronk PhD, Suzanne F.M. Van Wijck MD, Esther M.M. Van Lieshout PhD MSc, Michael H.J. Verhofstad MD PhD, Jonne T.H. Prins MD PhD, Mathieu M.E. Wijffels MD PhD, Suzanne Polinder PhD (on behalf of the FixCon study group)

Correspondence: Inge Spronk, Erasmus MC, Department of Public Health, i.spronk@erasmusmc.nl
