# Supplementary material for: Rib Fixation for Multiple Rib Fractures: Healthcare Professionals Perceived Barriers and Facilitators to Clinical Implementation
Source: World J Surg. 2023 Apr 4;47(7):1692–703. doi: 10.1007/s00268-023-06973-y (PMC10229739; doi:10.1007/s00268-023-06973-y)
Supplement: Supplementary file 3 — Supplementary file3 (DOCX 43 kb) [file 268_2023_6973_MOESM3_ESM.docx]

# **Online Resource 3. Comparison between different subgroups of healthcare professionals of barriers and facilitators influencing the implementation of surgical stabilization of rib fractures for multiple rib fractures after blunt trauma in adults**

|  |  | **Specialism** | | | | | | | |  | **Surgeons** | | | | | |
| --- | --- | --- | --- | --- | --- | --- | --- | --- | --- | --- | --- | --- | --- | --- | --- | --- |
|  |  | **Surgeons (n=32)** | |  | **Non-surgical physicians (n=19)** | |  | **Residents (n=10)** | |  | **FixCon center**  **(n=14)** | | |  | **Non-FixCon center (n=18)** | |
| **No.** | **Questionnaire item** | **(totally) disagree (%)** | **(totally) agree (%)** |  | **(totally) disagree (%)** | **(totally) agree (%)** |  | **(totally) disagree (%)** | **(totally) agree (%)** |  | **(totally) disagree (%)** | **(totally) agree (%)** | |  | **(totally) disagree (%)** | **(totally) agree (%)** |
|  | **Indication for SSRF** | | | |  |  | | |  |  |  |  | |  |  |  |
| 1 | I am aware in which patients, according to the current literature, rib fixation is indicated for multiple rib fractures (+) | **3.1** | **71.9** |  | **63.2** | **21.1** |  | **10.0** | **50.0** |  | 7.1 | 71.4 | |  | 0 | 72.2 |
| 2 | The indication for rib fixation of multiple rib fractures after blunt trauma is based on sound scientific knowledge (+) | 31.3 | 31.3 |  | 31.6 | 15.8 |  | 10.0 | 10.0 |  | 14.3 | 35.7 | |  | 44.4 | 27.8 |
| 3 | Relevance for the patient: I find rib fixation a suitable procedure for adults with multiple rib fractures caused by blunt trauma (+) | 9.4 | 59.4 |  | 10.5 | 52.6 |  | 20.0 | 30.0 |  | 0 | 71.4 | |  | 16.7 | 50.0 |
| 4 | The indication for rib fixation provides me with the opportunity to make my own consideration (+) | **3.1** | **75.0** |  | **31.6** | **5.3** |  | **0** | **70.0** |  | 7.1 | 78.6 | |  | 0 | 72.2 |
| 5 | The indication for rib fixation provides me with the opportunity to include the values of the patient (+) | **15.6** | **50.0** |  | **15.8** | **15.8** |  | **0** | **70.0** |  | 14.3 | 64.3 | |  | 16.7 | 38.9 |
| 6 | Knowledge: I have sufficient knowledge to assess who will benefit from rib fixation for multiple rib fractures (+) | **12.5** | **68.8** |  | **63.2** | **15.8** |  | **30.0** | **30.0** |  | 14.3 | 64.3 | |  | 11.1 | 72.2 |
| 7 | I have sufficient experience to assess who will benefit from rib fixation for multiple rib fractures (+) | **15.6** | **62.5** |  | **57.9** | **15.8** |  | **60.0** | **10.0** |  | 21.4 | 50.0 | |  | 11.1 | 72.2 |
| 8 | I am aware of the current literature on which level rib fixation can be beneficial (+) ^a^ | 31.3 | 43.8 |  |  |  |  |  |  |  | 50.0 | 35.7 | |  | 16.7 | 50.0 |
| 9 | I am aware of how to perform rib fixation on different locations on the rib (e.g. parasternal or paravertebral) (+) ^a^ | 31.3 | 53.1 |  |  |  |  |  |  |  | 50.0 | 42.9 | |  | 16.7 | 61.1 |
|  | **Concept and experience with rib fixation** **for multiple rib fractures** | | | | |  |  |  |  |  |  |  | |  |  |  |
| 10 | Completeness: The manufacturer’s rib fixation hardware provides the necessary operative instructions and materials (+) ^a^ | 3.1 | 62.5 |  |  |  |  |  |  |  | 7.1 | 50.0 | |  | 0 | 72.2 |
| 11 | Compatibility: Rib fixation of multiple rib fractures is compatible with how I am used to working with other indications and procedures (+) | **9.4** | **59.4** |  | **10.5** | **10.5** |  | **20.0** | **70.0** |  | 0 | 64.3 | |  | 16.7 | 55.6 |
| 12 | Complexity: I have sufficient skills to perform rib fixation (+) ^a^ | 15.6 | 75.0 |  |  |  |  |  |  |  | **35.7** | **57.1** | |  | **0** | **88.9** |
|  | Outcome: I perform rib fixation for multiple rib fractures to achieve the following goals for my patients (+): |  |  |  |  |  |  |  |  |  |  |  | |  |  |  |
| 13 | a. Pain relief | 9.4 | 71.9 |  | 5.3 | 57.9 |  | 0 | 100 |  | 7.1 | 78.6 | |  | 11.1 | 66.7 |
| 14 | b. Facilitate normal breathing | **3.1** | **90.6** |  | **5.3** | **57.9** |  | **0** | **100** |  | 7.1 | 85.7 | |  | 0 | 94.4 |
| 15 | c. Enhance lung capacity | 15.6 | 53.1 |  | 10.5 | 42.1 |  | 10.0 | 20.0 |  | 7.1 | 71.4 | |  | 22.2 | 38.9 |
| 16 | d. Recontour the chest wall | 12.5 | 53.1 |  | 5.3 | 42.1 |  | 30.0 | 40.0 |  | 14.3 | 35.7 | |  | 11.1 | 66.7 |
| 17 | e. Improve satisfaction with the recovery | 18.8 | 34.4 |  | 0 | 52.6 |  | 10.0 | 50.0 |  | 7.1 | 50.0 | |  | 27.8 | 22.2 |
| 18 | Satisfaction: In general, my patients will be satisfied when I perform rib fixation for multiple rib fractures (+) | **12.5** | **53.1** |  | **5.3** | **0** |  | **10.0** | **30.0** |  | 21.4 | 57.1 | |  | 5.6 | 50.0 |
| 19 | Support: I can rely on sufficient support from my colleagues if I need help with rib fixation for multiple rib fractures (+)^a^ | 3.1 | 93.8 |  |  |  |  |  |  |  | 7.1 | 92.9 | |  | 0 | 94.4 |
| 20 | Motivation to comply: I value the opinion of my close colleagues about rib fixation for multiple rib fractures (+) | **6.3** | **90.6** |  | **5.3** | **42.1** |  | **0** | **100** |  | 14.3 | 85.7 | |  | 0 | 94.4 |
|  | Normative beliefs: The following colleague or person expects me to perform rib fixation for multiple rib fractures (+) |  |  |  |  |  |  |  |  |  |  |  | |  |  |  |
| 21 | - Pulmonologist | 21.9 | 15.6 |  | 21.1 | 15.8 |  | 40.0 | 0 |  | **14.3** | **35.7** | |  | **27.8** | **0** |
| 22 | - (Trauma) surgeon | 6.3 | 65.6 |  | 10.5 | 36.8 |  | 0 | 80.0 |  | 7.1 | 78.6 | |  | 5.6 | 55.6 |
| 23 | - Thoracic surgeon | 18.8 | 9.4 |  | 15.8 | 15.8 |  | 30.0 | 10.0 |  | 28.6 | 14.3 | |  | 11.1 | 5.6 |
| 24 | - Patient with multiple rib fractures | 18.8 | 37.5 |  | 15.8 | 21.1 |  | 30.0 | 20.0 |  | **7.1** | **71.4** | |  | **27.8** | **11.1** |
| 25 | - Critical care specialist/ Intensivist | **6.3** | **65.6** |  | **15.8** | **31.6** |  | **30.0** | **20.0** |  | 7.1 | 78.6 | |  | 5.6 | 55.6 |
| 26 | - Anesthetist | 18.8 | 31.3 |  | 26.3 | 21.1 |  | 40.0 | 10.0 |  | 7.1 | 42.9 | |  | 27.8 | 22.2 |
|  | The following colleague or person expects me **not** to perform rib fixation for multiple rib fractures (-) |  |  |  |  |  |  |  |  |  |  |  | |  |  |  |
| 27 | - Pulmonologist | 31.3 | 6.3 |  | 10.5 | 15.8 |  | 50.0 | 10.0 |  | 35.7 | 7.1 | |  | 27.8 | 5.6 |
| 28 | - (Trauma) surgeon | 46.9 | 15.6 |  | 21.1 | 10.5 |  | 50.0 | 0 |  | 64.3 | 7.1 | |  | 33.3 | 22.2 |
| 29 | - Thoracic surgeon | 37.5 | 6.3 |  | 21.1 | 15.8 |  | 50.0 | 10.0 |  | 50.0 | 0 | |  | 27.8 | 11.1 |
| 30 | - Patient with multiple rib fractures | **40.6** | **3.1** |  | **10.5** | **15.8** |  | **70.0** | **0** |  | 50.0 | 0 | |  | 33.3 | 5.6 |
| 31 | - Critical care specialist/ Intensivist | 43.8 | 6.3 |  | 21.1 | 15.8 |  | 40.0 | 10.0 |  | 57.1 | 0 | |  | 33.3 | 11.1 |
| 32 | - Anesthetist | 37.5 | 6.3 |  | 15.8 | 21.1 |  | 50.0 | 10.0 |  | 50.0 | 7.1 | |  | 27.8 | 5.6 |
|  | Advantages and disadvantages of **rib fixation** **for multiple rib fractures** | | | | | | | | |  |  |  | |  |  |  |
| 33 | Personal benefits: Rib fixation for multiple rib fractures supports me to provide better care for my patients (+) | 6.3 | 56.3 |  | 10.5 | 42.1 |  | 0 | 70.0 |  | **7.1** | **78.6** | |  | **5.6** | **38.9** |
| 34 | Personal benefits: Rib fixation for multiple rib fractures increases my workload (-) | **62.5** | **12.5** |  | **15.8** | **52.6** |  | **50.0** | **10.0** |  | 57.1 | 21.4 | |  | 66.7 | 5.6 |
| 35 | Outcomes expectations: Rib fixation shortens the ICU length of stay (+) | 12.5 | 71.9 |  | 15.8 | 57.9 |  | 10.0 | 60.0 |  | 7.1 | 92.9 | |  | 16.7 | 55.6 |
| 36 | Outcomes expectations: Rib fixation shortens the hospital length of stay (+) | 18.8 | 53.1 |  | 10.5 | 57.9 |  | 10.0 | 70.0 |  | **7.1** | **71.4** | |  | **27.8** | **38.9** |
| 37 | Outcomes expectations: Rib fixation increases the pressure on the surgical schedule (-) | 18.8 | 53.1 |  | 5.3 | 73.7 |  | 20.0 | 70.0 |  | 14.3 | 42.9 | |  | 22.2 | 61.1 |
| 38 | Outcomes expectations: Rib fixation reduces the number of patients needing mechanical ventilation (+) | 15.6 | 53.1 |  | 15.8 | 52.6 |  | 20.0 | 30.0 |  | 7.1 | 71.4 | |  | 22.2 | 38.9 |
| 39 | Outcomes expectations: Rib fixation reduces pulmonary complications (+) | 12.5 | 56.3 |  | 15.8 | 63.2 |  | 10.0 | 60.0 |  | 14.3 | 64.3 | |  | 11.1 | 50.0 |
| 40 | Outcomes expectations: Rib fixation improves the quality of life of the patient (+) | 12.5 | 40.6 |  | 10.5 | 52.6 |  | 0 | 50.0 |  | 14.3 | 57.1 | |  | 11.1 | 27.8 |
| 41 | Outcomes expectations: Rib fixation reduces medical costs (+) | 43.8 | 6.3 |  | 36.8 | 15.8 |  | 40.0 | 10.0 |  | 35.7 | 7.1 | |  | 50.0 | 5.6 |
| 42 | Outcomes expectations: Rib fixation increases the number of visits to the outpatient clinic (-) | 40.6 | 9.4 |  | 15.8 | 21.1 |  | 30.0 | 10.0 |  | 35.7 | 0 | |  | 44.4 | 16.7 |
|  | **Organizational aspects of applying rib fixation** **for multiple rib fractures** | | | | | | | | |  |  | | | | | |
| 43 | Formal ratification by management: The management of my institution needs to make a formal policy about rib fixation (in management plans, protocols, etc.) (+) | 28.1 | 50.0 |  | 5.3 | 78.9 |  | 10.0 | 80.0 |  | **7.1** | **57.1** | |  | **44.4** | **44.4** |
| 44 | Coordination: It is necessary to have one or multiple persons assigned to coordinate the implementation of rib fixation in my institution (+) | 28.1 | 65.6 |  | 0 | 78.9 |  | 20.0 | 60.0 |  | 21.4 | 64.3 | |  | 33.3 | 66.7 |
| 45 | Unsettled organization: Other current or expected changes influence the implementation of rib fixation (for example, a reorganization, merger, budget cuts, staffing changes, or other innovations) (-) | 53.1 | 12.5 |  | 31.6 | 21.1 |  | 20.0 | 0 |  | 50.0 | 14.3 | |  | 55.6 | 11.1 |
| 46 | Replacement: There is a continuum of expertise to facilitate the implementation of rib fixation for multiple rib fractures (+) | 3.1 | 87.5 |  | 15.8 | 68.4 |  | 0 | 60.0 |  | 7.1 | 92.9 | |  | 0 | 83.3 |
| 47 | Staff: The current staff is sufficiently available for correctly implementing rib fixation for multiple rib fractures (+) | **6.3** | **87.5** |  | **21.1** | **47.4** |  | **0** | **70.0** |  | 14.3 | 78.6 | |  | 0 | 94.4 |
| 48 | Support available: There is sufficient financial and material support for correctly implementing rib fixation for multiple rib fractures (+) | **6.3** | **75.0** |  | **5.3** | **15.8** |  | **0** | **70.0** |  | 7.1 | 64.3 | |  | 5.6 | 83.3 |
| 49 | Time available: My institution allows me sufficient time to integrate rib fixation for multiple rib fractures in my daily clinical practice (+) | **3.1** | **84.4** |  | **10.5** | **15.8** |  | **0** | **70.0** |  | 0 | 85.7 | |  | 5.6 | 83.3 |
| 50 | Information accessible: Knowledge and experience concerning the implementation of rib fixation for multiple rib fractures are easily accessible in my institution (+) | **9.4** | **90.6** |  | **26.3** | **42.1** |  | **0** | **70.0** |  | 7.1 | 92.9 | |  | 11.1 | 88.9 |
| 51 | Feedback: My department provides feedback regularly about the implementation of rib fixation for multiple rib fractures (+) | **28.1** | **59.4** |  | **57.9** | **5.3** |  | **20.0** | **50.0** |  | 21.4 | 78.6 | |  | 33.3 | 44.4 |
| 52 | The COVID19 pandemic prohibits me from performing rib fixation for multiple rib fractures (-) | **78.1** | **3.1** |  | **21.1** | **5.3** |  | **30.0** | **20.0** |  | 64.3 | 7.1 | |  | 88.9 | 0 |
|  | **Other potential barriers to rib fixation** **for multiple rib fractures** | | | | | | | | |  |  |  |  | |  |  |
| 53 | The lack of evidence about the effectiveness hinders me from implementing rib fixation for multiple rib fractures (-) | **18.8** | **65.6** |  | **5.3** | **36.8** |  | **10.0** | **50.0** |  | **35.7** | **42.9** | |  | **5.6** | **83.3** |
| 54 | The lack of evidence about the **cost-**effectiveness hinders me from implementing rib fixation for multiple rib fractures (-) | 21.9 | 50.0 |  | 5.3 | 36.8 |  | 20.0 | 60.0 |  | 21.4 | 35.7 | |  | 22.2 | 61.1 |
| 55 | The considerable risk of postoperative complications hinders me from implementing rib fixation for multiple rib fractures (-) | **59.4** | **25.0** |  | **10.5** | **15.8** |  | **40.0** | **10.0** |  | 57.1 | 14.3 | |  | 61.1 | 33.3 |
| 56 | I am convinced that fixation for multiple rib fractures effectively improves the outcomes for my patients (+) | 15.6 | 37.5 |  | 15.8 | 42.1 |  | 20.0 | 50.0 |  | **7.1** | **64.3** | |  | **22.2** | **16.7** |
| 57 | I am convinced that fixation for multiple rib fractures is **cost**-effective (+) | 21.9 | 25.0 |  | 26.3 | 21.1 |  | 20.0 | 10.0 |  | 21.4 | 35.7 | |  | 22.2 | 16.7 |
| 58 | A practical guideline or advice from the Netherlands Association of Trauma surgery would stimulate me to implement rib fixation for multiple rib fractures (+) | 12.5 | 62.5 |  | 0 | 57.9 |  | 0 | 90.0 |  | 14.3 | 57.1 | |  | 11.1 | 66.7 |
| 59 | Expert centers should perform rib fixation for multiple rib fractures (+) | 43.8 | 31.3 |  | 26.3 | 47.4 |  | 40.0 | 40.0 |  | 42.9 | 35.7 | |  | 44.4 | 27.8 |

Note: (+) indicates positive statement; (-) indicates negative statement. Data are shown as percentages. Barriers are highlighted in red; facilitators are highlighted in blue. Bold numbers indicate statistically significant differences between subgroups.

^a^ Indicates that the question applies exclusively to surgeons.

Manuscript title: Rib fixation for multiple rib fractures: healthcare professionals perceived barriers and facilitators to clinical implementation

Journal: World Journal of Surgery

Authors: Inge Spronk PhD, Suzanne F.M. Van Wijck MD, Esther M.M. Van Lieshout PhD MSc, Michael H.J. Verhofstad MD PhD, Jonne T.H. Prins MD PhD, Mathieu M.E. Wijffels MD PhD, Suzanne Polinder PhD (on behalf of the FixCon study group)

Correspondence: Inge Spronk, Erasmus MC, Department of Public Health, i.spronk@erasmusmc.nl
